# Antihypertensive Effect of the GaMiSamHwangSaSimTang in Spontaneous Hypertensive Rats

**DOI:** 10.1155/2015/802368

**Published:** 2015-10-11

**Authors:** Kyungjin Lee, Bumjung Kim, Heseung Hur, Khanita Suman Chinannai, Inhye Ham, Ho-Young Choi

**Affiliations:** Department of Herbology, College of Korean Medicine, Kyung Hee University, 26 Kyungheedae-ro, Dongdaemun-gu, Seoul 130-701, Republic of Korea

## Abstract

The present study was designed to evaluate the antihypertensive effect of GaMiSamHwangSaSimTang (HVC1), a 30% ethanol extract of a mixture comprising Pruni Cortex, Scutellariae Radix, Coptidis Rhizoma, and Rhei Rhizoma, on spontaneous hypertensive rats (SHRs). The systolic blood pressure (SBP) was measured every 4 or 7 days using the noninvasive tail cuff system. The vasorelaxant effects on isolated aortic rings were evaluated. Aortic rings were contracted using phenylephrine (PE) or KCl, and the changes in tension were recorded via isometric transducers connected to a data acquisition system. In this study, oral administration of HVC1 decreased the SBP of SHRs over the experimental period. HVC1 induced concentration-dependent relaxation in the aortic rings that had been precontracted using PE or KCl. The vasorelaxant effects of HVC1 on endothelium-intact aortic rings were inhibited by pretreatment with *Nω*-Nitro-l-arginine methyl ester (L-NAME) or methylene blue. HVC1 inhibited the contraction induced by extracellular Ca^2+^ in endothelium-denuded rat aortic rings that had been precontracted using PE or KCl. In conclusion, HVC1 reduced the SBP of SHR and relaxed isolated SHR aortic rings by upregulating NO formation and the NO-cGMP pathway and blocking the entry of extracellular Ca^2+^ via receptor-operative Ca^2+^ channel and voltage-dependent Ca^2+^ channel.

## 1. Introduction

Hypertension is a global public health issue and is associated with increased risk of cardiovascular disease, stroke, and kidney disease [1]. The disease is regarded as a “silent killer” as it rarely produces symptoms in its early stages and as a result many people go undiagnosed [1]. In 2008, the worldwide prevalence of high blood pressure was reported to be approximately 40% of adults aged 25 or older; every year, about 9.4 million deaths are estimated to be caused by hypertension, which accounts for 12.8% of the worldwide total [1]. Hypertension has a huge economic impact, and in higher-income countries, including those in Eastern Europe and Central Asia, the disease accounts for almost 23% of health care expenditure [2].

In 2013, in Korea, the prevalence of hypertension in men and women over 30 years of age was 32.4 and 22.2%, respectively; the prevalence increased with age and in adults over 70 years of age, the prevalence of hypertension in men was 59% and in women was 64.3% [3]. The number of hypertensive patients is constantly increasing, and the cost of hypertension treatment is also steadily increasing in Korea. According to the National Health Insurance Statistical Yearbook 2013, the costs paid by national health insurance for the treatment of hypertension ($1.9 billion) formed a larger proportion of total medical costs ($46.3 billion) than any other disease, and—among 13.7 million patients—hypertension was the most common disease (5.5 million people) [4]. However, traditional medicines have not been widely used for the treatment of hypertension in Korea. There are several useful traditional medicines for the treatment of hypertension [5–7], but few patients choose to use them, and health insurance does not pay for them. This is because the herbal medicines for hypertension have not yet been developed throughout efficacy and safety studies in Korea.

Scutellariae Radix (SR), Coptidis Rhizoma (CR), and Rhizoma Rhei (RR) have been commonly used traditional medicines for cardiovascular diseases in China, Japan, and Korea. A herbal prescription SanHuangXieXinTang (SamHwangSaSimTang in Korean) composed of SR, CR, and RR was reported to decrease U46619-induced increase in pulmonary arterial blood pressure [8]. And we found that Pruni Cortex (PC) has potent vasorelaxant activities in the previous study [9]. Therefore, these herbal materials are expected to be useful for the treatment of spontaneous hypertension. For the development of new antihypertensive herbal medicine, a new prescription GaMiSamHwangSaSimTang (HVC1) which consists of four kinds of traditional medicine including Pruni Cortex, Scutellariae Radix, Coptidis Rhizoma, and Rhei Rhizoma was developed based on SamHwangSaSimTang. In the previous animal study, we found that vasorelaxant effect of HVC1 was better than SamHwangSaSimTang (data not shown).

In this study, we aimed to demonstrate the hypotensive effect and mechanisms of action of a HVC1 on spontaneous hypertensive rats and we performed standardization of HVC1.

## 2. Materials and Methods

### 2.1. Preparation of GaMiSamHwangSaSimTang

The extract was prepared from a mixture of dried PC (200 g), SR (100 g), CR (100 g), and RR (200 g). PC and RR were purchased from Dongwoodang Co., Ltd. (Yeongcheon, Kyungpook, Republic of Korea). CR and SR were purchased from Dong Yang Herb Co., Ltd. (Seoul, Republic of Korea). Professor Hocheol Kim of Kyung Hee University identified these herbal medicines. The mixture was extracted with 30% ethanol for 2 h in a reflux apparatus. After reflux and filtration, the extract was evaporated using a rotary vacuum evaporator (N-N series, EYELA, Japan) at 60°C and lyophilized to yield 159.9 g of crude extract.

### 2.2. Chemicals and Drugs

Phenylephrine (PE), KCl, acetylcholine, *Nω*-Nitro-l-arginine methyl ester (L-NAME), methylene blue (MB), atropine, indomethacin, and ethylene glycol-bis-(2-aminoethylether)-N,N,N′,N′-tetraacetic acid were purchased from Sigma Aldrich (St. Louis, MO, USA). Amlodipine besylate was purchased from Kolmar Korea Co., Ltd. (Yeongi-gun, Chungnam, Republic of Korea). All other reagents were of analytical purity.

### 2.3. Animals

Spontaneous hypertensive rats (SHR/lzm; male; weight: 200–250 g; age: 8 weeks) were purchased from Japan SLC Inc. (Hamamatsu, Shizuoka Prefecture, Japan). All procedures involving animals were conducted according to the animal welfare guidelines issued by the National Veterinary Research & Quarantine Service and World Organization for Animal Health (OIE), and this study was approved (KHUASP(SE)-13-018) by the Kyung Hee University Institutional Animal Care and Use Committee. The rats were housed under controlled conditions (22 ± 2°C; lighting, 07:00–19:00), and food and water were available* ad libitum*.

### 2.4. Measurement of Blood Pressure

The systolic blood pressure of SHRs was evaluated using the noninvasive tail cuff system (CODA 8-Channel High Throughput Non-Invasive Blood Pressure system, Kent Scientific Co. Ltd., Torrington, CT, USA) [10]. We randomly divided SHRs into four groups of six animals each. For 50 days, they were orally administered either distilled water, amlodipine, or HVC1. Amlodipine and HVC1 were dissolved in distilled water. Amlodipine was not completely soluble in distilled water; therefore, an aqueous suspension of amlodipine was used in this experiment. Control rats were treated with distilled water (1 mL·kg^−1^·day^−1^), positive control rats were treated with amlodipine (10 mg·kg^−1^·day^−1^), and the rats of the two experimental groups were treated with HVC1 (50 or 300 mg·kg^−1^·day^−1^).

### 2.5. Vasoactivity Measurement

Spontaneous hypertensive rat aortic rings were isolated and placed in organ chambers containing Krebs-Henseleit solution (K-H solution; 10 mL) at 37°C, and then the vasoactivity of HVC1 was evaluated using previously described methods [9]. HVC1 was dissolved in K-H solution. The endothelium-intact and endothelium-denuded aortic rings were contracted using PE (1 *μ*M) or KCl (60 mM) treatment. Endothelium-intact aortic rings were also preincubated with L-NAME (10 *μ*M), MB (10 *μ*M), indomethacin (10 *μ*M), or atropine (1 *μ*M) for 20 min before contraction with PE (1 *μ*M) treatment to investigate the vasorelaxant mechanisms of HVC1 action. The presence of functional endothelium was verified by the ability of ACh (10 *μ*M) to induce more than 80% relaxation in rings that were precontracted by PE (1 *μ*M). The relaxant effect of HVC1 on the aortic rings was calculated as a percentage of the contraction in response to PE or KCl.

### 2.6. High Performance Liquid Chromatography (HPLC) Analysis of HVC1

Precisely weighed HVC1 (100 mg) was dissolved in methanol (10 mL; HPLC grade; J. T. Baker Co. Ltd., USA) and the solution was filtered through a 0.45 *μ*m syringe filter (poly(vinylidene difluoride), Milford, USA). The analytical standards used for the HPLC analysis of HVC1 were as follows: sennoside A and sennoside B (Rhei Rhizoma standards, Sigma, USA), coptisine, berberine, wogonin (Rhizoma Coptidis standards, Sigma, USA), baicalin, baicalein (Coptidis Rhizoma standards, Sigma, USA), prunetin (Pruni Cortex standards, Sigma, USA), genistein-7-glucose, and prunetin-5-glucose (Pruni Cortex standards, isolated according to a previously published procedure [11]). Each standard (1 mg) was dissolved in 100 *μ*g/mL 50% methanol. Equal amounts of each standard mixture were combined and a HPLC chromatogram was obtained. The HPLC apparatus was a Gilson System equipped with a 234 Autosampler, a UV/VIS-155 detector, and a 321 HPLC Pump (Gilson, Seoul, Korea). A Luna 4.60 × 264 mm C18 reverse-phase column with 5 *μ*m particles (Phenomenex, CA, USA) was used. The mobile phase consisted of 0.1% formic acid (A) and acetonitrile (HPLC grade, J. T. Baker Co. LTD., USA) (B) in a ratio specified by the following binary gradient with linear interpolation: 0 min, 20% B; 60 min, 30% B; 70 min, 60% B; 100 min, 70% B. The column eluent was monitored at 250 nm, and all solvents were degassed with a micro-membrane filter (poly(tetrafluoroethylene), Advantec, Tokyo, Japan). Chromatography was performed at room temperature at a flow rate of 0.5 mL/min, using 10 *μ*L analyte, for 100 min.

### 2.7. Statistical Analysis

Data are expressed as mean ± standard error of the mean (SEM). Statistical comparisons were made using Student's *t*-test or one-way analysis of variance (ANOVA) followed by Tukey's post hoc test. All statistical analyses were performed using SPSS v.13.0 statistical analysis software (SPSS Inc., USA). *P* values less than 0.05 were considered statistically significant.

## 3. Results

### 3.1. Effect of HVC1 on Blood Pressure in SHRs

Before the experiment commenced (day 0), the systolic blood pressure (SBP) of the control group (198.8 ± 4.6 mmHg), the amlodipine 10 mg·kg^−1^·day^−1^ treated group (197.5 ± 1.4 mmHg), the HVC1 50 mg·kg^−1^·day^−1^ treated group (201.7 ± 1.5 mmHg), and the HVC1 300 mg·kg^−1^·day^−1^ treated group (203.3 ± 4.8 mmHg) were measured using the noninvasive tail cuff system. At the end of the experiment (day 50), the SBP of the control group had increased to 225.2 ± 1.9 mmHg. The SBP of the positive control group (i.e., the amlodipine-treated group) continuously decreased during the experimental period and thus this experiment was considered to be reliable.

**Figure 1:**
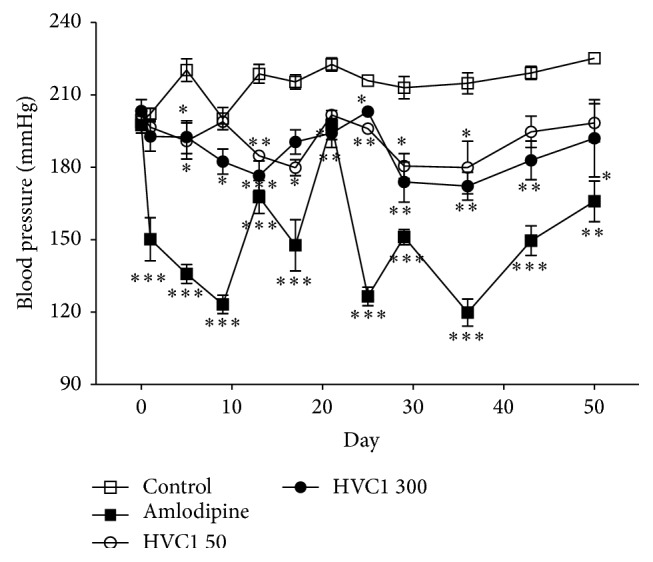
Hypotensive effect of HVC1 on the arterial systolic blood pressure of spontaneous hypertensive rats. Arterial systolic blood pressure was measured using the noninvasive tail cuff system. Values are expressed as mean ± SEM (*n* = 6). ^∗^ *P* < 0.05; ^∗∗^ *P* < 0.01; ^∗∗∗^ *P* < 0.001 versus control. Amlodipine = amlodipine 10 mg·kg^−1^·day^−1^ oral administration; HVC1 50 = HVC1 50 mg·kg^−1^·day^−1^ oral administration; HVC1 300 = HVC1 300 mg·kg^−1^·day^−1^ oral administration.

Orally administered HVC1 doses of 50 and 300 mg·kg^−1^·day^−1^ also continuously decreased the SBP of SHRs during the experimental period. On average, orally administered HVC1 doses of 50 and 300 mg·kg^−1^·day^−1^ decreased the SBP of SHRs to 191.1 ± 2.5 and 186.6 ± 2.9 mmHg, respectively. The maximal hypotensive effect was recorded on day 36 and SBP was 179.8 ± 10.8 and 172.2 ± 5.8 mmHg for the 50 and 300 mg·kg^−1^·day^−1^ dose-treated groups, respectively (Figure 1).

### 3.2. Effects of HVC1 on PE- or KCl-Induced Contraction of Endothelium-Intact or Endothelium-Denuded Aortic Rings

**Figure 2:**
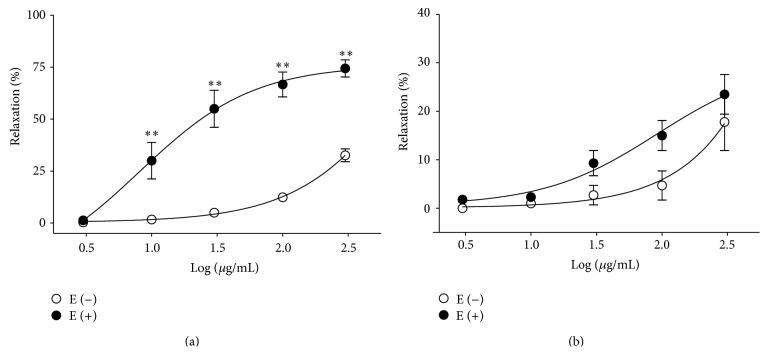
Concentration-dependent relaxant effects of HVC1 on precontracted spontaneous hypertensive rat aortic rings. Endothelium-intact [E (+)] or endothelium-denuded [E (−)] aortic rings were precontracted by phenylephrine (PE, 1 *μ*M) (a) or KCl (60 mM) (b). Values are expressed as mean ± SEM (*n* = 8). ^∗∗^ *P* < 0.01 versus E (−).

HVC1 (3, 10, 30, 100, and 300 *μ*g/mL) caused concentration-dependent relaxation in both endothelium-intact and endothelium-denuded aortic rings that had been precontracted using PE (1 *μ*M) treatment. However, endothelium-intact aortic rings were more relaxed than endothelium-denuded aortic rings (Figure 2(a)). HVC1 (3, 10, 30, 100, and 300 *μ*g/mL) also caused concentration-dependent relaxation in endothelium-intact and endothelium-denuded aortic rings that had been precontracted using KCl (60 mM) treatment. But there were no significantly differences between endothelium-intact and endothelium-denuded aortic rings (Figure 2(b)).

### 3.3. Effect of HVC1 on Endothelium-Intact Aortic Rings Preincubated with L-NAME or MB

**Figure 3:**
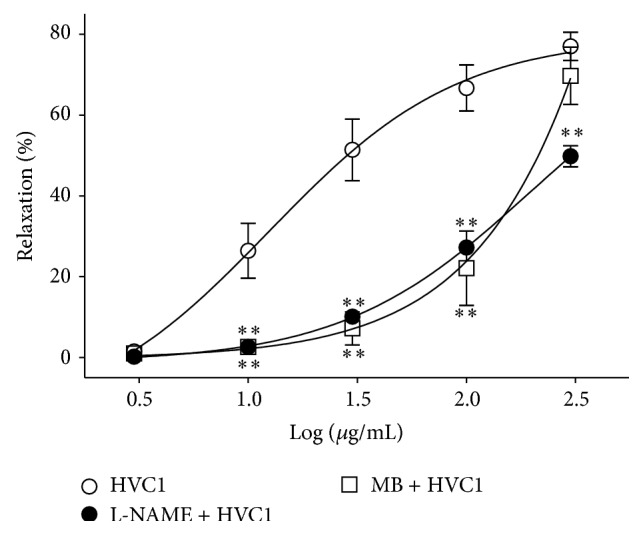
Effect of HVC1 on endothelium-intact aortic rings preincubated with *Nω*-Nitro-l-arginine methyl ester (L-NAME, 10 *μ*M) or methylene blue (MB, 10 *μ*M). The graph shows the relaxation responses induced by HVC1 in endothelium-intact spontaneous hypertensive rat aortic rings that had been precontracted with phenylephrine (PE, 1 *μ*M) in the presence or absence of L-NAME or MB in Krebs-Henseleit solution. The relaxant effects of HVC1 on isolated spontaneous hypertensive rat aortic rings were calculated as a percentage of the contraction in response to PE. Values are expressed as mean ± SEM (*n* = 4). ^∗^ *P* < 0.05 and ^∗∗^ *P* < 0.01 versus HVC1.

Incubation with L-NAME (10 *μ*M) or MB (10 *μ*M) significantly decreased HVC1-induced relaxation of endothelium-intact aortic rings that had been precontracted using PE (1 *μ*M) treatment. However, the vasorelaxant effect of HVC1 300 *μ*g/mL was not affected by preincubation with MB (Figure 3).

### 3.4. Effect of HVC1 on Endothelium-Intact Aortic Rings Preincubated with Indomethacin or Atropine

**Figure 4:**
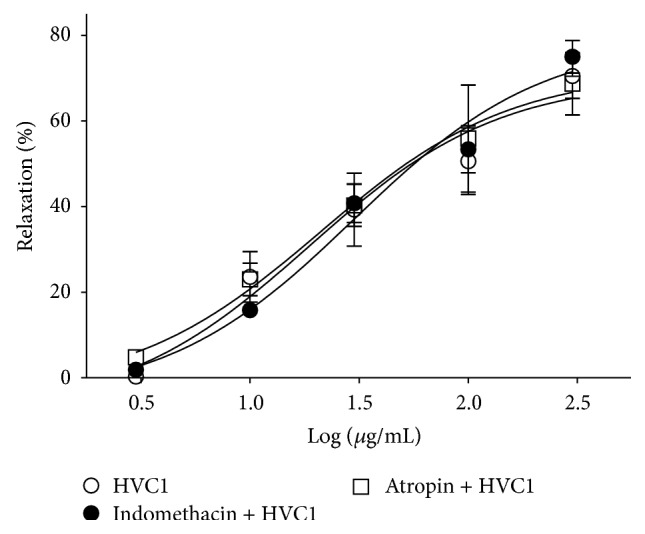
Effect of HVC1 on endothelium-intact aortic rings preincubated with indomethacin or atropine. Graph showing relaxation responses induced by HVC1 in endothelium-intact spontaneous hypertensive rat aortic rings that had been precontracted with phenylephrine (PE, 1 *μ*M) in Krebs-Henseleit solution in the presence or absence of indomethacin (10 *μ*M) or atropine (1 *μ*M). The relaxant effects of HVC1 on isolated spontaneous hypertensive rat aortic rings were calculated as a percentage of the contraction in response to PE. Values are expressed as mean ± SEM (*n* = 4).

Incubation with indomethacin (10 *μ*M) or atropine (1 *μ*M) did not affect HVC1-induced relaxation of endothelium-intact aortic rings that had been precontracted using PE (1 *μ*M) treatment (Figure 4).

### 3.5. Effect of HVC1 on Extracellular Ca^2+^-Induced Contraction

**Figure 5:**
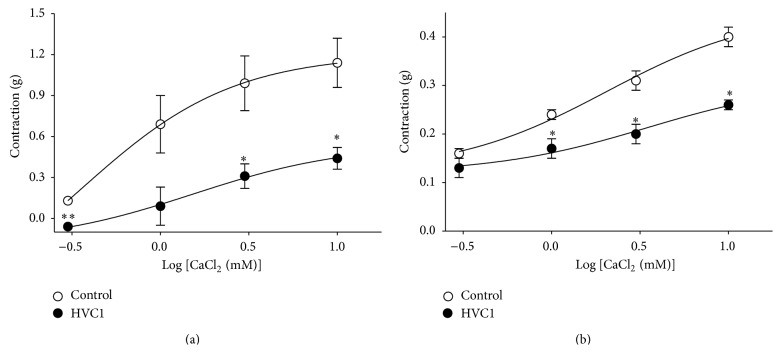
Effect of HVC1 on Ca^2+^-induced aortic ring contraction. Graph showing the inhibitory effect of HVC1 (300 *μ*g/mL) on the contraction induced by extracellular Ca^2+^ addition (0.3–10 mM) in endothelium-denuded spontaneous hypertensive rat aortic rings that had been precontracted using phenylephrine (PE, 1 *μ*M) (a) or KCl (60 mM) (b) treatment in Ca^2+^-free Krebs-Henseleit solution. Values are expressed as mean ± SEM (*n* = 4). ^∗^ *P* < 0.05 and ^∗∗^ *P* < 0.01 versus control.

In Ca^2+^-free K–H solution, the cumulative addition of CaCl_2_ (0.3–10 mM) induced progressively increased tension in rat aortic rings that had been precontracted using PE (1 *μ*M; Figure 5(a)) or KCl (60 mM; Figure 5(b)) treatment. As shown in Figure 5, preincubation with HVC1 (300 *μ*g/mL) for 20 min significantly inhibited the contraction induced by extracellular Ca^2+^.

### 3.6. Standard Material Analysis

**Figure 6:**
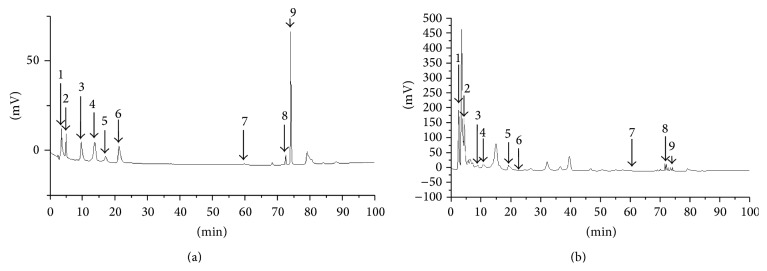
HPLC chromatogram of HVC1 standard mixtures (a) and HVC1 (b). 1: sennoside A; 2: genistein-7-O-*β*-glucopyranoside; 3: coptisine; 4: baicalin; 5: prunetin-5-O-*β*-glucopyranoside; 6: berberine; 7: baicalein; 8: wogonin; 9: prunetin.

The retention times of the standards in the sample mixture were as follows: sennoside A, 3.49 min; genistein-7-glucose, 4.98 min; coptisine, 9.61 min; baicalin, 13.78 min; prunetin-5-glucose, 17.18 min; berberine, 21.22 min; baicalein, 59.76 min; wogonin, 72.53 min; prunetin, 74.12 min (Figure 6(a)). In the HPLC chromatogram of HVC1, the peaks for the standards were observed (Figure 6(b)).

## 4. Discussion

In this study, HVC1, a herbal prescription containing extracts of PC, SR, CR, and RR, decreased the SBP of SHRs and relaxed aortic rings that had been contracted by treatment with PE or KCl.

Over the 50-day long experimental period, the SBP of the control group (orally administrated distilled water) increased from 198.8 ± 4.6 mmHg on day 0 to 225.2 ± 1.9 mmHg on day 50. On the other hand, the SBP of the HVC1 50 and 300 mg·kg^−1^·day^−1^ treated groups significantly decreased during the experimental period. These results suggest that HVC1 has an antihypertensive effect.

Vascular tone is important for the regulation of blood pressure. In blood vessels, the vascular endothelium and smooth muscle play an important role in vasorelaxation. The vascular endothelium releases potent vasodilators such as nitric oxide (NO) and prostacyclin (PGI_2_) [12].

NO is synthesized from l-arginine and when released from the vascular endothelium, it activates cyclic guanidine monophosphate (cGMP), which leads to relaxation of vascular smooth muscles [12]. Thus, NO synthesis and the cGMP pathway are important factors in hypertension. In this study, preincubation with L-NAME (10 *μ*M), an inhibitor of NO synthase, significantly decreased the HVC1-induced relaxation of endothelium-intact aortic rings that had been contracted using PE treatment. Preincubation with MB (10 *μ*M), a soluble guanylate cyclase inhibitor, also significantly decreased HVC1-induced relaxation. These results suggested that the antihypertensive and vasorelaxant effects of HVC1 are partly related to NO synthesis and the NO-cGMP pathway.

PGI_2_ is synthesized by cyclooxygenase and when released from the vascular endothelium, it activates adenyl cyclase (AC). Activated AC increases the intracellular concentration of cyclic adenosine monophosphate (cAMP), which relaxes vascular smooth muscle by decreasing the intracellular calcium concentration [12]. In this study, preincubation with indomethacin (10 *μ*M) did not affect the HVC1-induced relaxation of endothelium-intact aortic rings that had been contracted using PE treatment. These results suggested that PGI_2_ might not be involved in the antihypertensive and vasorelaxant effect of HVC1.

Muscarinic receptors located on endothelial or smooth muscle cells also play an important role in vasorelaxation [13]. In this work, preincubation with atropine (1 *μ*M), a nonselective muscarinic receptor antagonist, did not affect HVC1-induced relaxation of endothelium-intact aortic rings that had been contracted using PE treatment. This result suggested that the muscarinic receptor might not contribute to the antihypertensive and vasorelaxant effects of HVC1.

The contraction and relaxation of vascular smooth muscle can be regulated by extracellular Ca^2+^ influx via the receptor-operative Ca^2+^ channel (ROCC) or the voltage-dependent Ca^2+^ channel (VDCC) without endothelial derived factors [14]. In the present study, preincubation with HVC1 (300 *μ*g/mL) for 20 min significantly inhibited the contraction induced by extracellular Ca^2+^ supplementation in endothelium-denuded aortic rings that had been contracted using PE or KCl treatment in Ca^2+^-free K–H solution. These results suggested that the antihypertensive and vasorelaxant effects of HVC1 are partly related to blockage of extracellular Ca^2^ entry via the ROCC and VDCC.

Furthermore, HVC1 (300 *μ*g/mL) inhibited the PE-induced contractions to a greater extent than high K^+^-induced contractions (Figure 5). Several reports have described that the involvement of the contractile elements is more related to agonist-induced contractions than high K^+^. Phosphorylation of myosin light chain (MLC) is induced to a greater extent by receptor agonist like phenylephrine than by high K^+^ [15]. Moreover, drugs such as forskolin [16] and sodium nitroprusside [17] decrease the agonist-induced contractions to a greater extent than high K^+^-induced contractions owing to the involvement of the contractile elements. The agonists like phenylephrine cause an initial spike in Ca^2+^ followed by small sustained rise in Ca^2+^ above the basal levels, thus increasing the Ca^2+^ sensitivity of MLC phosphorylation and leading to increased contraction. However, high K^+^ depolarization results in a maintained increase in the Ca^2+^-induced contractions [18]. These findings suggest that HVC1 involves the contractile elements of the aortic smooth muscle cells suggesting one of the possible mechanisms that HVC1 selectively inhibits the receptor-linked Ca^2+^ channel or that it decreases the Ca^2+^ influx or Ca^2+^ sensitivity.

HVC1 is a herbal prescription consisting of PC, SR, CR, and RR extracts. Sennoside A is a known standard component of RR, baicalin, baicalein, and wogonin are known standard components of SR, and berberine is a known standard component of CR [19]. However, the standard components of PC have not yet been established. In a previous study, we isolated genistein-7-*O*-*β*-glucopyranoside and prunetin-5-*O*-*β*-glucopyranoside from* Prunus* bark [11]. Prunetin is known as one of the major components of the* Prunus* species [20]. Therefore, we used these compounds as standards in the HPLC analysis of HVC1. Among these compounds, coptisine [21], baicalin [22, 23], berberine [24, 25], and wogonin [26] showed vasorelaxant activities and we found that prunetin also has vasorelaxant activities (data not shown). Therefore, these compounds might be the active compounds in HVC1 that help treat hypertension.

## 5. Conclusions

In this study, HVC1 reduced the SBP of SHRs and relaxed isolated SHR aortic rings by upregulating NO formation and the NO-cGMP pathway and blocking the entry of extracellular Ca^2+^ via ROCC and VDCC. Therefore, HVC1 could be a useful herbal medicine for the prevention and treatment of hypertension.
